# Use of Microwave Radiometry to Monitor Thermal Denaturation of Albumin

**DOI:** 10.3389/fphys.2018.00956

**Published:** 2018-07-25

**Authors:** Yuri Ivanov, Andrey F. Kozlov, Rafael A. Galiullin, Vadim Y. Tatur, Vadim S. Ziborov, Nina D. Ivanova, Tatyana O. Pleshakova, Sergey G. Vesnin, Igor Goryanin

**Affiliations:** ^1^Institute of Biomedical Chemistry, Moscow, Russia; ^2^Foundation of Advanced Technologies and Innovations, Moscow, Russia; ^3^Joint Institute for High Temperatures of Russian Academy of Sciences (RAS), Moscow, Russia; ^4^Moscow State Academy of Veterinary Medicine and Biotechnology, Moscow, Russia; ^5^RES LTD, Moscow, Russia; ^6^Medical MicroWave Radiometry (MMWR) LTD, Edinburgh, United Kingdom; ^7^School of Informatics, University of Edinburgh, Edinburgh, United Kingdom; ^8^Biological Systems Unit, Okinawa Institute of Science and Technology, Okinawa, Japan; ^9^Tianjin Institute of Industrial Biotechnology, Chinese Academy of Sciences, Tianjin, China

**Keywords:** water clusters, protein denaturation, rate constant, microwave emission, temperature

## Abstract

This study monitored thermal denaturation of albumin using microwave radiometry. Brightness Temperature, derived from Microwave Emission (BTME) of an aqueous solution of bovine serum albumin (0.1 mM) was monitored in the microwave frequency range 3.8–4.2 GHz during denaturation of this protein at a temperature of 56°C in a conical polypropylene cuvette. This method does not require fluorescent or radioactive labels. A microwave emission change of 1.5–2°C in the BTME of aqueous albumin solution was found during its denaturation, without a corresponding change in the water temperature. Radio thermometry makes it possible to monitor protein denaturation kinetics, and the resulting rate constant for albumin denaturation was 0.2 ± 0.1 min^−1^, which corresponds well to rate constants obtained by other methods.

## Introduction

In medical studies, microwave radiometry (MWR) is based on the intensity of intrinsic electromagnetic radiation of the patient's tissues in the microwave range (Barrett and Myers, 1975; Carr, 1989; Leroy et al., 1998). MWR has found wide application in medical diagnostics (Carr, 1989; Leroy et al., 1998; Han et al., 2001; Stauffer et al., 2013, 2014; Livanos et al., 2018). To date, commercially available equipment has been developed to allow early diagnosis of cancers and other diseases (Vesnin, 2010)^1^. In the field of biochemical research, MWR is just starting to evolve (Ivanov et al., 2016a,b).

In this paper, the possibility of using MWR to study kinetics of protein denaturation is demonstrated. The study of thermal denaturation of proteins is important for understanding protein stability and for modeling their physicochemical properties. We used MWR to monitor the denaturation of albumin, the best characterized protein. Human serum albumin (HSA) is the most abundant protein in human blood plasma. Genes encoding HSA reside on chromosome 4 (Nishio and Dugaiczyk, 1996). Measurements of HSA are very important for diagnosis and treatment of many diseases (Anderson, 2000; Green et al., 2012). Since this protein is highly homologous to Bovine Serum Albumin (BSA) (Both are stabilized with 17 disulfide bonds and have the same isoelectric point.), the latter is used in modeling the properties of HSA (Gelamo et al., 2002; Vetri et al., 2007). BSA contains 583 amino acids (Steinhardt et al., 1971; Hirayama et al., 1990; Gelamo et al., 2002; Moriyama et al., 2008; Ahmad et al., 2011) and has a molecular weight of 66.4 kDa. Its secondary structure consists mainly of alpha-helices. Denaturation and protein aggregation are generally investigated using scanning calorimetry and optical methods (Vetri et al., 2007; Borzova et al., 2016). The BSA denaturation rate constant at a temperature of 60°C is 0.5 min^−1^ (Borzova et al., 2016). Thus, using BSA denaturation as an example, this paper demonstrates the use of MWR in biochemical studies. Existing biochemical methods, including those used to study albumins (Steinhardt et al., 1971) typically have a number of disadvantages. Optical methods, such as circular dichroism methods require use of optical spectral instrumentation, which is quite expensive and may be difficult to operate. The drawbacks of calorimetry are the complexity and duration of the analysis. It should be noted, the advantages of using new methods in addition to known methods are that they provide more information about the denaturation processes under investigation.

In this work, new applications of this technique for measuring kinetics of albumin denaturation are demonstrated by monitoring the change in the brightness temperature in the microwave range. In the long term, development of MWR and its adaptation for studying other biochemical processes, could potentially result in a new analytical platform in biomedical research. MWR is easy to operate, does not require labels, and as noted earlier, can be used to study radiative properties of biological systems.

## Materials and methods

De-ionized, ultrapure water (Milli-Q System, Millipore, USA) was used for all solutions. Lyophilized bovine serum albumin BSA [Sigma] was dissolved in deionized water at a concentration of 0.1 mM, close to the concentration of albumin in vertebrate blood (Fasano et al., 2005). pH of BSA solution was 7.00 ± 0.05.

## Measurement of radiometric brightness in the microwave range

We used a microwave radiometer RTM-01 RES (www.mmwr.co.uk) to measure electromagnetic radiation in the microwave range. The power of electromagnetic radiation in the frequency range Δ*f* of the medium is proportional to the radiometric (brightness) temperature of the medium *T*_*rad*_ (Weisblat, 2003). For the microwave range, the power of the microwave signal at the antenna output can be represented as:

(1) $$\begin{array}{r} P=kT_{rad}\Delta f\left(1-R\right), \end{array}$$

where Δ*f* is the frequency band of the microwave radiometer, in GHz, and *k* is Boltzmann's constant, R is the reflection coefficient of antenna, P- is the microwave radiation power of the heated aqueous medium of the protein received by the antenna.

The antenna is designed in such a way as to minimize the reflection coefficient R. In addition, in the zero-balance radiometer used in the present studies, the measurement results are weakly dependent on the change in the reflection coefficient. Therefore, for balanced zero radiometers, *R* = 0 is usually assumed. In accordance with eq. (1), by measuring the power of the radiation of the medium in the microwave range, it is possible to obtain information on the brightness *T*_*rad*_(Brightness Temperature derived from Microwave Emission, BTME), which characterizes the emissivity of the water-protein medium. The measurement range of 3.4–4.2 GHz is selected based on the results of our early experiments (Ivanov et al., 2016a,b). So, in this range we observed the radiation of a solution of enzyme biomolecules in the course of their functioning. The functioning is accompanied by the restructuring of the hydrate shells of bio-molecules, so we assumed that the melting processes of the protein can also lead to a rearrangement of its hydration envelope, and, consequently, to changes in the spectral characteristics of the water-protein medium in this frequency range. In addition, the convenience of using this range is determined by the fact that it operates commercial radiometers. We measured the BTME of water and the BTME of an aqueous solution of BSA in the microwave range 3.4–4.2 GHz. The error in measuring the BTME was ±0.1°C. To record radiation in the microwave range, a whip antenna connected to the microwave radiometer was used.

## Procedure to prepare solutions for measurement

Water and 0.1 mM aqueous protein solutions were heated using a thermostat (T-24, BIS, Russia) to a temperature of 56°C, after which the sample was taken by pipette with a volume of 1 mL. Measurements of BTME were carried out after the solution was injected into a conical polypropylene measuring cuvette with a conical base diameter of 25 mm and a height of 17 mm. The cuvette was inserted into a thermostat Thermomixer Comfort (Eppendorf, Germany). Measurements were carried out for 25–60 min. All measurements were repeated twice.

## Results

The control series of the experiments included measurements of BTME (t) of pure water and BSA solution at a temperature of 23°C, when BSA denaturation is not observed. In either case, there was no change in BTME (t) during the observation time of 22 min.

**Figure 1:**
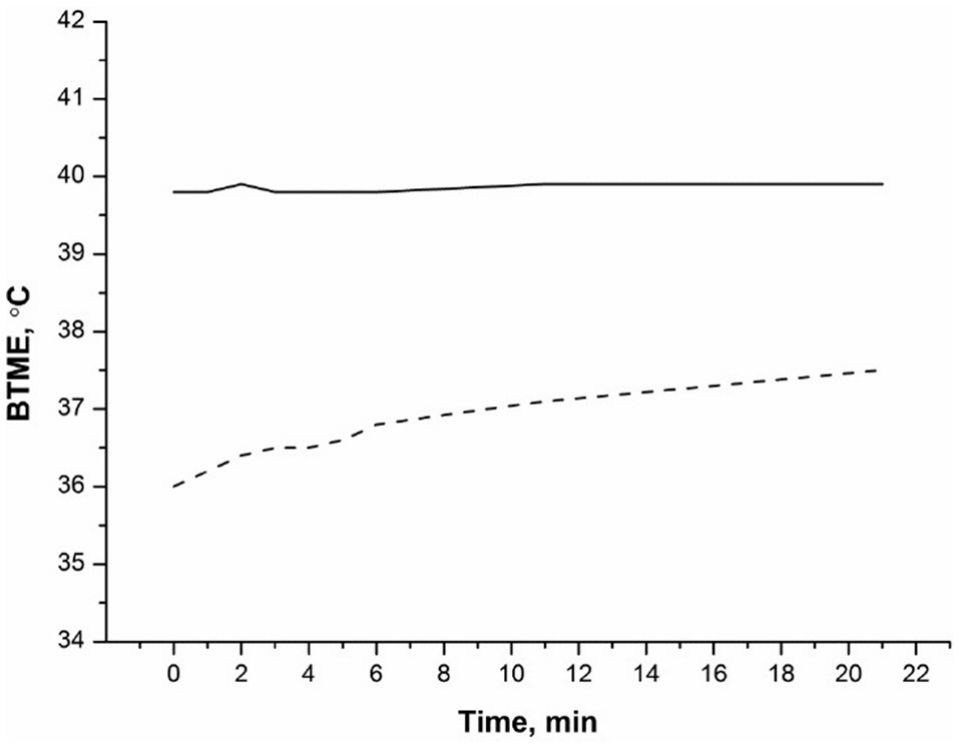
Kinetics of thermal denaturation of 0.1 mM BSA. BTME (BSA) (- - -), BTME (water) (__). The temperature of the solution was 56°C. Measurements of microwave radiation were performed using an MWR-01 RES radiothermometer.

Radiothermometry was used to monitor kinetics of thermal denaturation of BSA in water at a temperature of T = 56°C. We analyzed the BTME of an albumin solution in water, we have used the BTME of water as a control to exclude its possible effect on the results. During the observation time of 22 min, changes in the BTME albumin solution were observed (see Figure 1), while from 25 to 60 min of observation, water and the BSA solution were practically unchanged (therefore no data in this time range are presented). The BTME of water did not change significantly with time (Figure 1). At the same time, the BTME of BSA changed significantly. During the initial period, the BTME of BSA was less than the BTME of water at ~4°C. During an observation time of about 25 min, BTME of BSA increased on the order of 1.5–2 ° C, (Figure 1). Error of experiment was of the order of 0.2°C. The thermodynamic temperature of water and the albumin solution did not change during the experiment.

**Figure 2:**
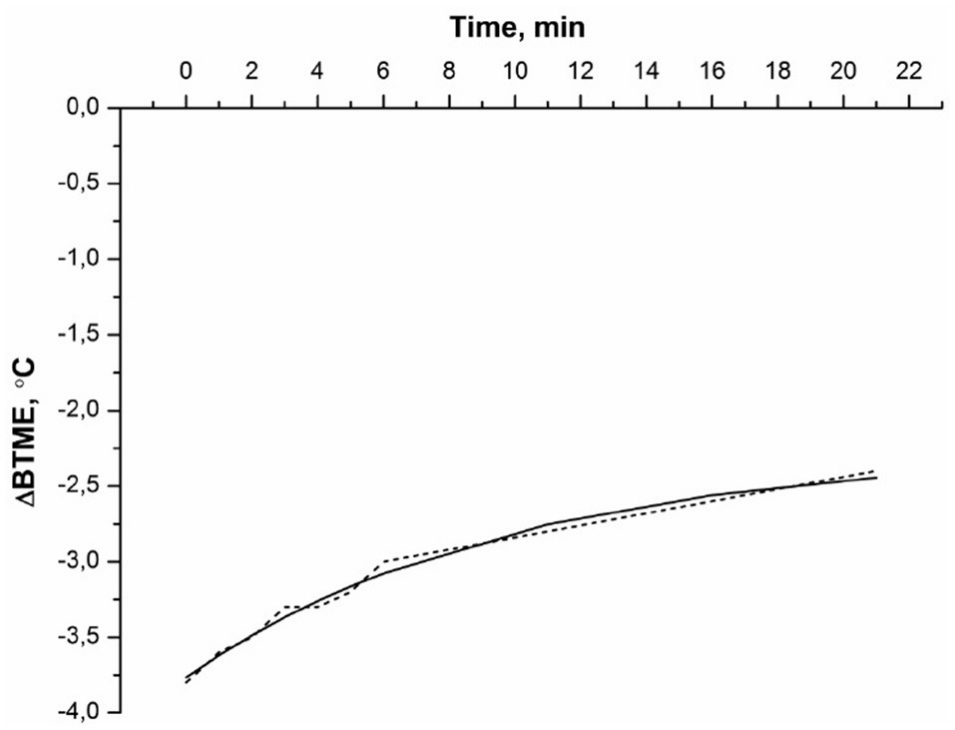
The difference between the BTME of BSA and that of water: ΔBTME (t) = BTME (BSA)—BTME (water). Experimental conditions were the same as in Figure 1. The experimental data (…), the approximation curve (___) according to eq. (2).

For better explanation of the experimental data, the effect of water was excluded, and a difference curve between the BTME of the protein solution and BTME of the water (control) was calculated. The difference between the BTME of BSA and that of water, ΔBTME (t) = BTME (BSA)–BTME (water), is shown in Figure 2.

The ΔBTME(t) of BSA decayed monotonically (Figure 2). This dependence was approximated by an empirical equation:

(2) $$\begin{array}{r} \Delta BTME\left(t\right)=Ae^{-k_{den}t}+B, \end{array}$$

where *A* is the pre-exponential factor, *k*_*den*_ is the denaturation rate constant, and *t* is the time of measurement.

The thermal denaturation curve of albumin (Figure 2) is well described by the exponential curve (2) with *k*_*den*_ = 0.10 min^−1^, where *A* = 1.49, *B* = −3.77. Approximation error BTME (t) does not exceed 0.2°C. Average value of *k*_*den*_ = (0.2 ± 0.1) min^−1^. Note that our data showing significant changes in BTME of BSA at 56°C is in agreement with experimental data derived from scanning calorimetry (Borzova et al., 2016), indicating that the BSA denaturation process should be observed in this temperature range. The denaturation rate constant at 60°C, determined using scanning calorimetry, was *k*_*den*_≅ 0.5 min^−1^ (Borzova et al., 2016). Thus, the BTME (t) of BSA solution determined using MWR is close to that value. The reason for the BTME change in albumin solution during its thermal denaturation is as follows. First, it is known that while BSA denatures, the ratios of ortho/para isomers of water change at the same time, as noted for chymotrypsin solution (Bunkin and Pershin, 2013). Second, as albumin denatures, it aggregates (Borzova et al., 2016), leading to a change in the ratio of ortho/para isomers of water (Pershin, 2013). The rotation spectrum of water isomers is in the GHz range (Bunkin and Pershin, 2013). These measurements are also made with a radiothermometer in the GHz band, so the denaturation of the protein leads to a change in the spectral properties of the solution within the sensitivity range of the instrument. Thus, MWR is well suited to monitoring protein denaturation.

MWR monitors the process of denaturation as a set of several processes described above, which allows refinement of models of protein denaturation. This method does not require any isotopes or fluorescent labels. It is simple and inexpensive compared with calorimetry and optical spectrometry and it allows quantum effects associated with the ratio of ortho/para isomers of water to be evaluated.

## Discussion

The work demonstrates that microwave radiometry allows direct monitoring of thermal denaturation of proteins, without the use of labels. This distinguishes it from complex methods using fluorescent labels or isotope tags. This method is much cheaper than circular dichroism or microcalorimetric methods. Within the microwave range, microwave radiometry makes it possible to monitor kinetics of protein denaturation associated with changes in these spectral properties of a medium containing a protein. These spectral properties are determined by the quantum-mechanical characteristics of this medium and provide information on the nature of the intermolecular interaction. Such characteristics include description the rotational transitions of water molecules to ortho- and para-states, the degree of disequilibrium of the aqueous medium in terms of its spin temperature, reflecting the complex heterostructure of water (Bunkin and Pershin, 2013). Moreover, some of the para-H2O molecules form hydrogen-bound complexes, for example, the structures of the hydrate shells of proteins (Pershin, 2013). The ratio of ortho/para isomers of pure water at constant temperature, as seen in Figure 1, does not change. However, in the presence of protein, the possible conversion of ortho/para isomers of water can be stimulated due to the change in the hydrate coat of the protein during its melting. The rotational transitions of hydroxy groups are in the GHz range, and the values of the detuning of the ortho/para states of the water molecules are also in this range (Pershin, 2013). In the same range, the spectral properties of the water-protein medium during the melting of the protein were measured. This allows us to suggest that changes in the brightness temperature of the medium measured in the range 3.4–4.2 GHz during the melting of the protein are associated with a change in the ratio ortho/para isomers an aqueous medium. Therefore, using this method, it is possible to observe quantum mechanical transitions of ortho/para isomers an aqueous medium in a protein solution during denaturation process. Accordingly, it is possible to obtain new information for the theoretical modeling of protein denaturation when the protein interacts with an aqueous microenvironment.

Microwave radiometry has a high potential to become a standard method for studying physico-chemical properties of protein media. This is because in addition to characterizing the absorbing properties of aqueous protein solutions, microwave radiometry makes it possible to determine the radiative properties of enzyme solutions (Ivanov et al., 2016a,b) and to monitor cellular functions in the microwave range (Zynov'ev and Vesnin, 2009; Ivanov et al., 2016c).

## Future applications in brain stroke and trauma

The brightness temperature of water and protein solutions at physiological temperatures (Zynov'ev and Vesnin, 2009; Ivanov et al., 2016d, 2017; Vesnin et al., 2017), should permit applications this method in diagnostics of human and animal hemodynamics and for describing processes of water balance in living systems. In clinics, MWR has been successfully used for breast cancer (Vesnin et al., 2017), Among other clinical applications, MWR has long been used to monitor brain damage (Butrov et al., 2012) after stroke and other brain injuries. In the future, microwave radiometry could have clinical applications in stroke and trauma.

## Author contributions

YI supervised the project. AK, RG, VT, VZ, and NI carried out the experiments. TP carried out the experiments and wrote the paper. SV provided equipment and consultancy. IG wrote the paper and provided consultancy.

### Conflict of interest statement

The authors declare that the research was conducted in the absence of any commercial or financial relationships that could be construed as a potential conflict of interest.

## Footnotes

^1^Localized Cancer Tumor Detection Using Microwaves and Nanoparticles. Patent US15617440 registered (2017)-06-08, Priority date 2016-03-15.
